# Zoledronic Acid Prevents Bone Resorption Caused by the Combination of Radium-223, Abiraterone Acetate, and Prednisone in an Intratibial Prostate Cancer Mouse Model

**DOI:** 10.3390/cancers15164115

**Published:** 2023-08-15

**Authors:** Mari I. Suominen, Matias Knuuttila, Birgitta Sjöholm, Timothy Wilson, Esa Alhoniemi, Dominik Mumberg, Sanna-Maria Käkönen, Arne Scholz

**Affiliations:** 1 Pharmatest Services Ltd., 20520 Turku, Finland; 2 Aurexel Life Sciences Ltd., 21240 Askainen, Finland; 3 Inoi Oy, 20100 Turku, Finland; 4 Research & Development, Pharmaceuticals, Bayer AG, 13353 Berlin, Germany; 5 Institute of Biomedicine, University of Turku, 20520 Turku, Finland

**Keywords:** radium-223, prostate, bone metastases, CRPC, zoledronic acid, bone-protecting agents

## Abstract

**Simple Summary:**

The majority of patients with advanced prostate cancer eventually develop bone metastases. Radium-223 has improved overall survival and relieved symptoms related to bone metastases in patients with metastatic castration-resistant prostate cancer (mCRPC). In a phase 3 trial (ERA 223), concomitant treatment with radium-223, abiraterone acetate, and prednisone increased the risk of non-pathological fractures in patients with mCRPC. Here, we evaluated the efficacy and potential bone health-related effects of radium-223, abiraterone acetate, prednisone, and zoledronic acid as monotherapies or combinations in an intratibial LNCaP prostate cancer xenograft model mimicking bone metastatic prostate cancer. Moreover, we identified mechanisms that may have potentially contributed to the increased fracture risk of patients with mCRPC treated with the combination of radium-223, abiraterone, and prednisone. These results may help understand the safety aspects of such combination treatments and prevent fractures associated with these combination treatments in the clinic.

**Abstract:**

An increased risk of non-pathological fractures in patients with prostate cancer and bone metastases has been associated with combination treatment with radium-223, abiraterone, and prednisone/prednisolone in the absence of bone-protecting agents. Here, we investigated possible mechanisms leading to this outcome using an intratibial LNCaP model mimicking prostate cancer bone metastases. Male NOD.scid mice were inoculated intratibially with LNCaP prostate cancer cells and treated with vehicle, radium-223, abiraterone, prednisone, zoledronic acid, or their combinations for 28 days. Serum TRACP 5b and PSA levels were measured. Bone structure, quality, and formation rate of non-tumor-bearing and tumor-bearing tibiae were analyzed by microCT, 3-point bending assay, and dynamic histomorphometry, respectively. Radium-223 incorporation into bone was also measured. Radium-223/abiraterone/prednisone combination treatment induced a transient increase in bone resorption indicated by elevated TRACP 5b levels, which was inhibited by concurrent treatment with zoledronic acid. Furthermore, radium-223/abiraterone/prednisone combination reduced periosteal and trabecular new bone formation and the number of osteoblasts, but bone structure or biomechanical quality were not affected. The abiraterone/prednisone treatment decreased radium-223 incorporation into tumor-bearing bone, possibly explaining the lack of additional antitumor efficacy. In conclusion, radium-223/abiraterone/prednisone combination increased bone resorption, which may have been one of the mechanisms leading to an increased fracture risk in patients with mCRPC.

## 1. Introduction

Radium-223 dichloride (radium-223, Xofigo^®^) is a targeted alpha therapy that homes to areas of increased bone turnover, such as bone metastases, and induces DNA double-strand breaks in cancer cells, osteoblasts, and osteoclasts [1,2]. Radium-223 is actively incorporated into bone by osteoblasts, but passive incorporation into bone mineral hydroxyapatite is also observed. To date, radium-223 is the only alpha-emitting radionuclide approved for the treatment of patients with metastatic castration-resistant prostate cancer (mCRPC). In a pivotal phase 3 trial (ALSYMPCA), radium-223 increased overall survival, delayed time to symptomatic skeletal events, and improved quality of life in patients with CRPC metastasized to bone [3].

The rationale for combining radium-223 with other approved drugs is to improve treatment outcomes by increasing survival and reducing the occurrence of skeletal-related events (SREs). To date, numerous clinical trials have evaluated various treatment combinations with radium-223. Most of these clinical trials have focused on combining radium-223 with abiraterone acetate (abiraterone), prednisone, enzalutamide, docetaxel, PARP inhibitors, or various immunotherapies to evaluate the efficacy and impact on bone health of concomitant treatment [4,5].

Promising results in combining radium-223 with abiraterone acetate and prednisone were obtained in a phase 2 trial (eRADicAte), showing a reduction in the number of bone lesions and clinically meaningful improvements in quality of life with decreased pain [6]. On the other hand, in a phase 3 trial (ERA 223), an increased risk of fractures was observed in patients with bone metastatic CRPC treated with radium-223 in combination with abiraterone and prednisone/prednisolone during the median follow-up of 21 months [7]. While overall survival or SRE-free survival did not significantly differ between the study arms, fractures were observed in 29% of patients treated with the combination of radium-223, abiraterone, and prednisone compared with 11% of patients treated with the combination of abiraterone and prednisone [7]. However, concurrent administration of bone-protecting agents (BPAs), such as denosumab, zoledronic acid, or other bisphosphonates, seemed to reduce fractures in these patients [7]. Both zoledronic acid and denosumab are used in the clinic to prevent skeletal-related complications in men with CRPC and bone metastases [8,9]. Likewise, in the ongoing phase 3 trial PEACE III (NCT02194842), where enzalutamide monotherapy is compared to a combination of radium-223 and enzalutamide, the risk of bone fractures was almost abolished by mandatory administration of BPAs [10,11]. Recent real-world data studies have also reported lower fracture rates in patients with mCRPC receiving radium-223 and abiraterone or enzalutamide when they were concurrently treated with BPAs [12,13,14].

Here, we investigated the effects of radium-223, abiraterone, prednisone, zoledronic acid, and their combinations on bone using the intratibial LNCaP prostate cancer xenograft model. Using this model, the aim of this study was to investigate the plausible mechanisms explaining the increased risk of (non-pathological) fractures observed in the ERA 223 phase 3 trial. The intratibial LNCaP model recapitulates the abnormal osteoblastic bone growth and mixed osteoblastic/osteolytic lesions observed in prostate cancer bone metastases [15]. Thereby, the model mimics the tumor-induced changes in bone that are typical of metastatic prostate cancer. 

## 2. Materials and Methods

### 2.1. Compounds

Radium-223 dichloride (radium-223) was synthesized at Bayer AG and diluted with 28 mmol/L sodium citrate to form a solution with a concentration of 66 kBq/mL radium-223. The dosing volume of 5 mL/kg was used, resulting in a dose of 330 kBq/kg. For prednisone treatment, 60-day slow-release pellets (SG-161, Innovative Research of America, Sarasota, FL, USA) with a total amount of 2.5 mg of prednisone were subcutaneously implanted. Abiraterone acetate (abiraterone) (MedChemExpress EU, Sollentuna, Sweden) was dissolved in benzyl benzoate (5% of the final volume). The solution was stirred at +50 °C for 5 min and supplemented with peanut oil to obtain a final concentration of 20 mg/mL. The solution was stirred at +50 °C until dissolved. Zoledronic acid (0.04 mg/mL infusion solution, Medac GmbH, Wedel, Germany) was diluted with sterile 0.9% NaCl to a final concentration of 0.02 mg/mL. Sodium citrate (28 mmol/L) and 5% benzyl benzoate in peanut oil were used as vehicle. 

### 2.2. Cell Culture

LNCaP human prostate cancer cells (CRL-1740™, ATCC) were cultured in standard cell culture conditions (+37 °C, 5% CO_2_) until semiconfluent and authenticated using short tandem repeat analysis (GenePrint10 system, Promega, Madison, WI, USA) at the Institute for Molecular Medicine Finland (FIMM, Helsinki, Finland). The cells were tested negative for mycoplasma contamination in-house and for murine pathogens by IDEXX Laboratories Inc. (Ludwigsburg, Germany).

### 2.3. Animals

Male NOD.scid immunodeficient mice (NOD.CB17/*Prkdc^scid/scid^*, Janvier Labs/Envigo) were housed in individually ventilated cages and fed an irradiated soy-free diet (Teklad Global Diets 2916) and UV-purified tap water *ad libitum*. The acclimatization period for the mice was 5 days. Animal experiments were conducted with the approval of the Animal Experiment Board in Finland (license number: ESAVI-2331-04 10 07-2017), according to the guidelines of the European Union directive 2010/63/EU.

### 2.4. Intratibial LNCaP Model

LNCaP human prostate cancer cells (2 × 10^6^ in 20 μL of PBS) were inoculated into the bone marrow cavity of the right proximal tibia of 5–7 weeks old male mice (NOD.scid). For intratibial inoculations and imaging, the mice were anesthetized with inhalation of isoflurane (IsoFlo vet 100%, Zoetis Finland Oy, Helsinki, Finland). Analgesia was provided with buprenorphine (Temgesic^®^, 0.3 mg/mL, Indivior Europe Ltd., Dublin, Ireland): 0.1 mg/kg, s.c. before inoculation, and 0.02 mg/mL in drinking water for 2 days after inoculation. The animals were weighed twice weekly and at sacrifice to monitor the progression of the disease. Any abnormal clinical signs were recorded. The first measurements of prostate-specific antigen (PSA) were performed 10 days before the dosing was started, approximately 6 weeks after the cell inoculation. The mice were stratified into treatment groups (*n* = 9–14 mice/group) based on serum PSA and tartrate-resistant acid phosphatase 5b (TRACP 5b) levels and total bone volume of the tumor-bearing tibia as measured by in vivo micro-computed tomography (microCT). The procedures are described in detail in the Supplementary Materials.

**Figure 1:**
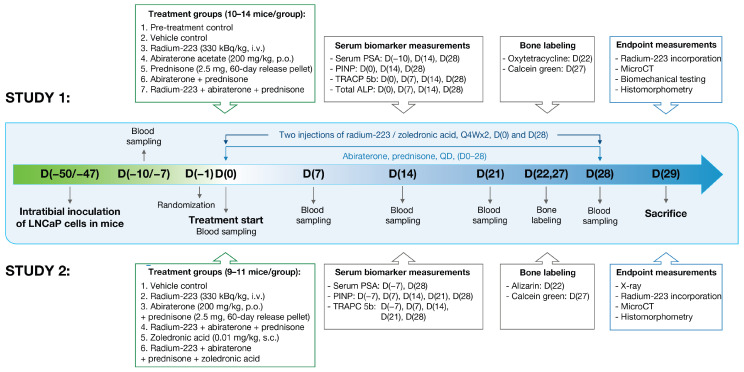
The study outline and timeline of the intratibial LNCaP xenograft model. Approximately seven weeks after inoculation, the mice in two separate studies (Study 1 and 2) were stratified to treatment groups and treated with vehicle, radium-223 (300 kBq/kg, Q4Wx2, i.v.), abiraterone acetate (200 mg/kg, QDx28, p.o.), prednisone (2.5 mg/kg, 60-day release pellet), zoledronic acid (0.01 mg/kg, Q4Wx2, s.c.) or different combination treatments for 28 days. D(number) indicates days after treatment started.

Two separate in vivo studies (Study 1 and Study 2) were conducted, in which the mice were treated with vehicle, radium-223 (330 kBq/kg, Q4Wx2, i.v.), abiraterone (200 mg/kg, QDx28, p.o.), prednisone (2.5 mg, 60-day release pellet) and zoledronic acid (0.01 mg/kg, Q4Wx2, s.c.) as a monotherapy or as combinations of abiraterone and prednisone; radium-223, abiraterone, and prednisone; or radium-223, abiraterone, prednisone, and zoledronic acid, for 28 days (Figure 1).

At the end of the studies, the mice were sacrificed by CO_2_ asphyxiation followed by cervical dislocation. At necropsy, all macroscopic findings were recorded. Both tibiae and the left or right femur were measured for length and weight and collected into liquid scintillation vials containing cold 40% EtOH for measurements of radium-223 incorporation. The other femur was stored at −20 °C for biomechanical quality analyses. 

### 2.5. Blood Sampling and Biomarkers

Before blood sampling, the mice were fasted for 4 h. The blood samples (100–200 μL) from the saphenous vein of mice were collected into Microvette 100 Z and 200 Z-Gel tubes (Sarstedt Ag and Co, Nümbrecht, Germany). The tubes were gently inverted, and the blood was allowed to clot at room temperature for 30–60 min, followed by centrifugation at 10,000× *g* at room temperature for 5 min. Serum samples were collected and stored at −80 °C for biomarker analyses. 

Serum PSA samples were taken either every two weeks or at the end of the study, depending on the study (Figure 1). The serum samples for bone resorption markers TRACP 5b and C-terminal telopeptide of type I collagen (CTX-I), and bone formation markers procollagen type I N-terminal propeptide (PINP) and total alkaline phosphatase (ALP) were collected once weekly or every two weeks after the treatment start. Serum PSA and TRACP 5b levels were measured using the Human PSA (R&D Systems, Minneapolis, MN, USA) and MouseTRAP^®^ (IDS Ltd., Boldon, UK) ELISA assays, respectively. Serum PINP, CTX-I, and ALP levels were measured using the Rat/Mouse PINP EIA (IDS Ltd.), RatLaps® (CTX-I) EIA (IDS Ltd.), and Colorimetric Alkaline Phosphatase Assay Kit (Abcam, Cambridge, UK), respectively. All biomarker assays were analyzed using a VICTOR2 Multilabel Counter (PerkinElmer, Waltham, MA, USA).

### 2.6. Ex Vivo Radiography

At sacrifice, the tumor-bearing tibiae were imaged using a Faxitron Specimen Radiographic System MX-20 D12 (Faxitron Corp., Lincolnshire, IL, USA) and Faxitron Dicom software version 3.0. Tumor-induced abnormal bone area in tumor-bearing tibiae was determined by drawing the outlines of radiopaque and radiolucent areas and analyzed using MetaMorph image analysis software version 7.8.0.0 (Molecular Devices LLC, Sunnyvale, CA, USA).

### 2.7. Radium-223 Incorporation to Bone

In order to assess the incorporation of radium-223 into bone, the radioactivity of non-tumor-bearing and tumor-bearing tibiae and the left or right femur were measured using an automatic gamma counter (Hidex, Turku, Finland). In addition, the diaphysis, epiphysis, and metaphysis of the femur were measured separately.

### 2.8. Bone Structure, Quality, and Formation Measurements

The bone volume, cross-sectional dimensions, and bone structure of non-tumor-bearing and tumor-bearing tibiae were quantified using a SkyScan 1276 high-resolution microCT scanner (Bruker, Billerica, MA, USA) [16,17,18]. The biomechanical quality of the femoral shafts was measured by a 3-point bending test using an Instron 3343 biomechanical testing system (Instron, Norwood, MA, USA) [19,20,21,22,23]. Bone microarchitecture, bone formation, and cellular characteristics were analyzed by bone histomorphometry using an OsteoMeasure7 histomorphometry system (OsteoMetrics, Decatur, GA, USA) [24,25]. All parameters were analyzed following the guidelines of the American Society for Bone and Mineral Research (ASBMR) [26], including the reported parameters in Table S1. For bone labeling, injections of oxytetracycline (20 mg/kg, i.p.) or alizarin (30 mg/kg, s.c.) and calcein green (10 mg/kg, s.c.) were given to mice seven and two days before sacrifice, respectively. The methods are described in detail in the Supplementary Materials.

### 2.9. Histology

Tumor area, cortical and trabecular bone areas, and fibrotic and necrotic areas of tumor-bearing tibiae were analyzed from histology sections using Pannoramic 250 Flash and Pannoramic 1000 slide scanners (3DHISTECH Ltd., Budapest, Hungary). The methods are described in detail in the Supplementary Materials.

### 2.10. Statistical Analyses

Statistical analyses were performed with the R statistical software version 3.3 [27]. Longitudinal serum biomarker data were either square root- (PSA), log- (PINP, TRACP 5b), or non-transformed (ALP) and analyzed using a liner mixed-effects model, and the comparisons were carried out using model contrasts, except in Study 2, in which PSA values relative to baseline were analyzed using ANOVA, and the pairwise comparisons were performed using ANOVA contrasts. For ex vivo radiography analysis (total area of abnormal bone), the data were transformed using square root transform and analyzed using ANOVA contrasts. Endpoint data (bone structure, quality, formation) were analyzed using ANOVA followed by contrasts, or Kruskal–Wallis test and Dunn’s test if the assumptions of ANOVA were not met. Kruskal–Wallis test and pairwise comparisons using Dunn’s test were applied for histology analyses. Radium-223 incorporation data were analyzed using either Student’s *t*-test or ANOVA. The obtained *p* values were adjusted for multiple comparisons.

## 3. Results

### 3.1. Combination Treatment with Radium-223, Abiraterone, and Prednisone Has No Additive Effects on Tumor Growth but Reduces Radium-223 Incorporation into Bone

**Figure 2:**
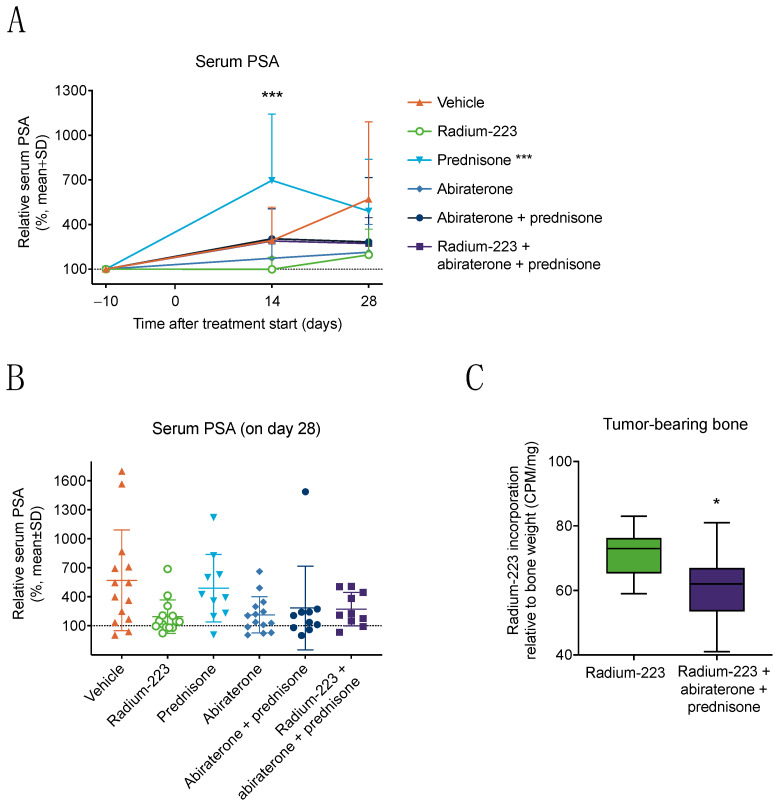
Combination treatment with radium-223, abiraterone, and prednisone has no additive effects on tumor growth but reduces radium-223 incorporation into bone. Blood samples were collected from the saphenous vein of LNCaP tumor-bearing mice (*n* = 10–14) treated with vehicle, radium-223 (330 kBq/kg, Q4Wx2), prednisone (2.5 mg, 60-day release pellet), abiraterone (200 mg/kg, QD), or their combinations. Relative serum PSA levels presented (**A**) during the study and (**B**) at the end of the study. The serum PSA levels were measured by ELISA and normalized to pre-treatment baseline values. Statistical analyses in panel A were performed using mixed models and model contrasts: ***, *p* < 0.001, compared with vehicle on day 14. (**C**) Radium-223 incorporation into tumor-bearing bones (only tibia) collected from mice treated with radium-223 monotherapy (*n* = 14) or the combination of radium-223, abiraterone, and prednisone (*n* = 11). The results are expressed as counts per minute (CPM) normalized to the weight of the bone sample. The box plot (**C**) describes median, 25/75% quartiles, and the minimum and maximum values. Statistical analyses were performed using Student’s *t*-test: *, *p* < 0.05.

In vivo antitumor efficacy of radium-223 with abiraterone and prednisone was investigated using the intratibial LNCaP prostate cancer xenograft model. The efficacy of treatments was monitored by measuring serum PSA levels. An increase in tumor burden was observed in vehicle-treated mice, as indicated by increasing PSA levels (Figure 2A). No differences in PSA levels were observed between radium-223 monotherapy and the combination of abiraterone and prednisone or the combination of radium-223, abiraterone, and prednisone at the end of the study (Figure 2B), indicating that combining radium-223 with abiraterone and prednisone has no additive effect on tumor growth suppression. However, a transient increase in serum PSA levels was observed after two weeks of prednisone monotherapy compared with vehicle treatment (*p* < 0.001) (Figure 2A). Furthermore, body weights remained stable throughout the study, with less than a 20% decline observed in the relative body weight. A decreasing trend in body weight was observed in all groups receiving prednisone (Figure S1).

As radium-223 is a radioactive isotope that seeks and accumulates into areas of active bone metabolism, radium-223 incorporation into bone tissue was investigated by measuring the radioactivity of bone samples using an automatic gamma counter. Radium-223 incorporation in tumor-bearing bone was reduced by the combination treatment of radium-223, abiraterone, and prednisone compared with radium-223 monotherapy (*p* = 0.028) (Figure 2C).

### 3.2. Radium-223, Abiraterone, and Prednisone Combination Treatment Transiently Increases Bone Resorption

**Figure 3:**
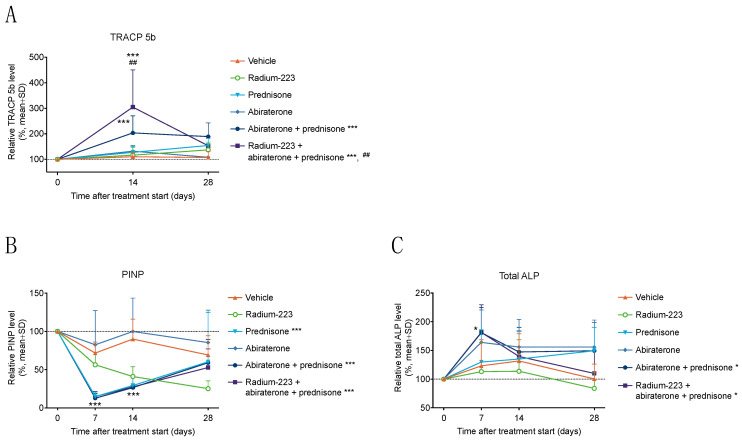
Radium-223, abiraterone, and prednisone combination treatment transiently increases bone resorption. Relative (**A**) TRACP 5b, (**B**) PINP, (**C**) total ALP levels were measured in blood samples collected from the saphenous vein of LNCaP tumor-bearing mice (*n* = 10–14) 7, 14 or 28 days after the initiation of treatment and normalized to pre-treatment baseline values. The mice were treated with vehicle, radium-223 (330 kBq/kg, Q4Wx2), prednisone (2.5 mg, 60-day release pellet), abiraterone (200 mg/kg, QD), or their combinations. Statistics were performed using mixed models, and model contrasts on day 7 (PINP, total ALP) and on day 14 (TRACP 5b, PINP): *, *p* < 0.05; ***, *p* < 0.001, compared with vehicle; ^##^, *p* < 0.01, compared with abiraterone and prednisone combination.

To examine bone turnover, bone turnover biomarkers, including bone formation markers PINP and total ALP and bone resorption marker TRACP 5b, were measured in serum samples from LNCaP tumor-bearing mice. TRACP 5b levels were clearly increased by the radium-223, abiraterone, and prednisone combination treatment during the first two weeks of treatment compared with the combination treatment of abiraterone and prednisone (*p* = 0.005) but returned close to the baseline level at sacrifice (Figure 3A and Figure S2A). Compared with the vehicle treatment, both the combination treatment of radium-223, abiraterone, and prednisone (*p* < 0.001) and, to a lesser extent, the combination of abiraterone and prednisone (*p* < 0.001) increased TRACP 5b 14 days after treatment initiation (Figure 3A and Figure S2A). In contrast to an increase of TRACP 5b, a radical decrease in PINP was observed in all groups receiving prednisone (*p* < 0.001) compared with the vehicle treatment during the first two weeks (Figure 3B and Figure S2B). After 4 weeks of treatment, PINP levels returned to a level comparable to the vehicle treatment. Radium-223 monotherapy decreased PINP levels gradually during the treatment period, and PINP levels were not affected by abiraterone monotherapy (Figure 3B and Figure S2B). Total ALP levels demonstrated a rapid peak after one week of abiraterone monotherapy (*p* = 0.29), the abiraterone and prednisone combination (*p* = 0.016), and the radium-223, abiraterone, and prednisone combination (*p* = 0.017) (Figure 3C and Figure S2C). Together, these results indicate that the radium-223, abiraterone, and prednisone combination treatment transiently increased bone resorption. 

### 3.3. Radium-223, Abiraterone, and Prednisone Combination Treatment Shows No Treatment-Specific Effects on Bone Structure or Biomechanical Quality

**Figure 4:**
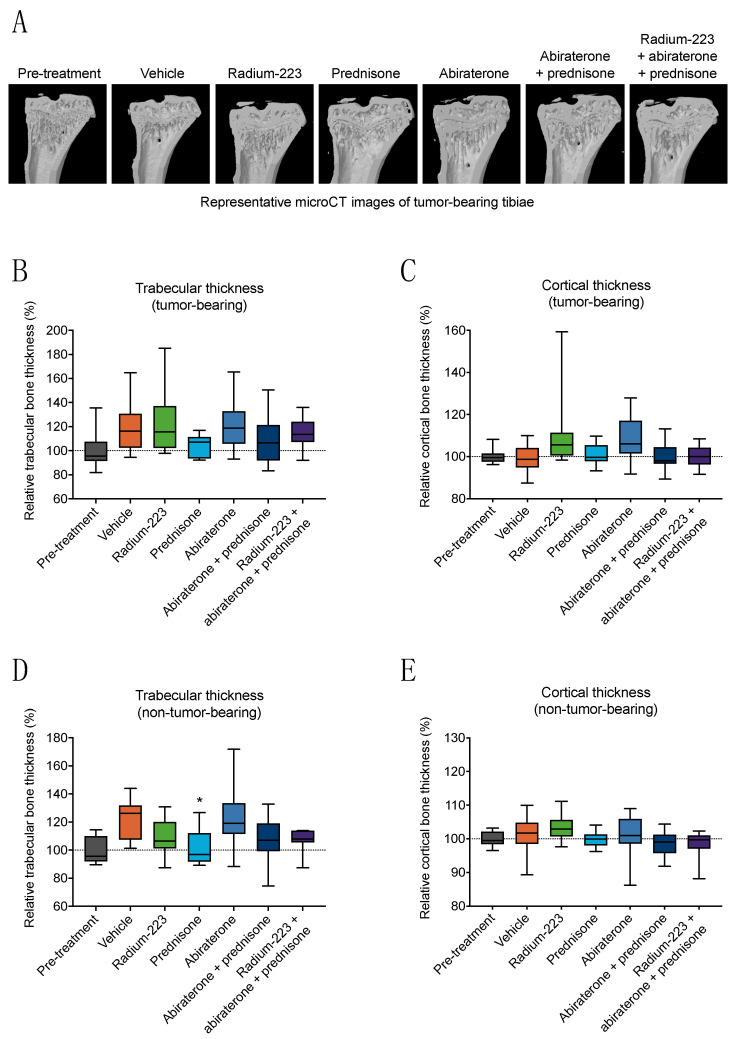
Radium-223, abiraterone, and prednisone combination treatment shows no treatment-specific effects on bone structure or biomechanical quality. The mice (*n* = 10–14) were treated with vehicle, radium-223 (330 kBq/kg, Q4Wx2), prednisone (2.5 mg, 60-day release pellet), abiraterone (200 mg/kg, QD) or their combinations. Bone thickness of non-tumor-bearing tibiae was quantified using a microCT scanner. (**A**) Representative microCT images of the longitudinal sections of tumor-bearing tibiae at sacrifice. Relative change in (**B**) trabecular thickness and (**C**) cortical thickness in tumor-bearing bone, and relative change in (**D**) trabecular thickness and (**E**) cortical thickness in non-tumor-bearing bone, from randomization to sacrifice as normalized to values at randomization. Statistical analyses were performed using Kruskal–Wallis test and Dunn’s test: *, *p* < 0.05, compared with vehicle. The box plots (**B**–**E**) describe median, 25/75% quartiles, and the minimum and maximum values.

Bone structural parameters in non-tumor-bearing and tumor-bearing tibiae, including bone volume and cross-sectional dimensions, were quantified using a microCT scanner (Figure 4A), whereas the biomechanical quality of femoral shafts was analyzed using a 3-point bending test. Neither trabecular thickness (Figure 4B) nor cortical thickness (Figure 4C) in tumor-bearing bone were affected in any of the treatment groups compared with the vehicle treatment. Prednisone monotherapy reduced trabecular thickness (*p* = 0.034) in non-tumor-bearing bone compared with the vehicle treatment (Figure 4D), while cortical thickness remained unaffected (Figure 4E). However, the combination of radium-223, abiraterone, and prednisone had no effect on trabecular or cortical thickness (Figure 4B–E). In addition, no treatment-specific effects for the combination of radium-223, abiraterone, and prednisone in non-tumor-bearing or tumor-bearing tibiae were observed in total, trabecular, or cortical bone volume (Figure S3A–F). In general, the effects of radium-223, abiraterone, or prednisone monotherapies were less prominent on the tumor-bearing bone (Figure S3B,D,F). Furthermore, no treatment-specific effects for the combination of radium-223, abiraterone, and prednisone were observed in biomechanical testing of non-tumor-bearing femoral shafts (Figure S4A–E).

### 3.4. Radium-223, Abiraterone, and Prednisone Combination Treatment Inhibits Both Trabecular and Cortical Bone Formation in Non-Tumor-Bearing Bone

**Figure 5:**
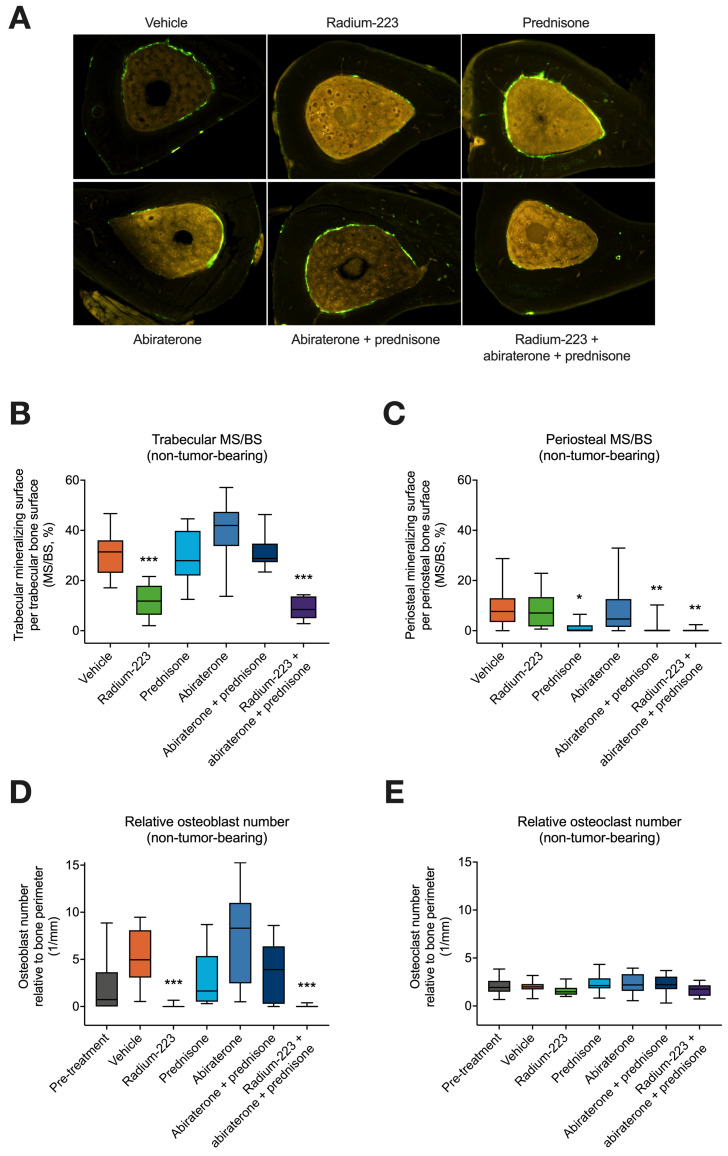
Radium-223, abiraterone, and prednisone combination treatment inhibits both trabecular and cortical bone formation in non-tumor-bearing bone. The mice were treated with vehicle, radium-223 (330 kBq/kg, Q4Wx2), prednisone (2.5 mg, 60-day release pellet), abiraterone (200 mg/kg, QD), or their combinations. (**A**) Representative cross-sectional images (4x magnification) of non-tumor-bearing tibiae as determined by histomorphometry. Bone formation was described as (**B**) trabecular and (**C**) periosteal mineralizing surface per trabecular and periosteal bone surface (MS/BS) in non-tumor-bearing tibiae, respectively. MS/BS describes the ratio of the mineralized surface on the trabecular/periosteal bone surface to the entire surface of the trabecular/periosteal bone. The number of (**D**) osteoblasts and (**E**) osteoclasts on the trabecular bone surface relative to the bone perimeter in non-tumor-bearing tibiae (*n* = 10–14). The bones were labeled with oxytetracycline and calcein green in vivo for measuring the dynamic histomorphometry parameters. Statistical analyses were performed using Kruskal–Wallis test and Dunn’s test: *, *p* < 0.05; **, *p* < 0.01; ***, *p* < 0.001, compared with vehicle. The box plots (**B**–**E**) describe median, 25/75% quartiles, and the minimum and maximum values.

Histomorphometry analyses were performed to quantify bone amount, microarchitecture, and cellular characteristics in non-tumor-bearing tibiae (Figure 5A). Trabecular mineralizing surface was reduced in all groups of LNCaP tumor-bearing mice receiving radium-223 (*p* < 0.001) (Figure 5B), while periosteal mineralizing surface in cortical bone was reduced in all groups receiving prednisone (*p* = 0.004–0.015) (Figure 5C). However, only the combination treatment of radium-223, abiraterone, and prednisone reduced both trabecular and periosteal mineralizing surface compared with the vehicle treatment (Figure 5A,B), indicating that the combination treatment reduces new cortical bone (*p* = 0.004) and trabecular bone (*p* < 0.001) formation in non-tumor-bearing bone. In line with the inhibition of bone formation, both the combination of radium-223, abiraterone, and prednisone (*p* < 0.001) and radium-223 monotherapy (*p* < 0.001) decreased the number of osteoblasts in non-tumor-bearing bone (Figure 5D), confirming the known direct effects of radium-223 radiation on osteoblast number [15]. No such changes were observed in the number of osteoclasts in non-tumor-bearing bone (Figure 5E). Furthermore, in the histological analyses of the tumor-bearing tibiae, no differences were observed between the treatment groups and the vehicle group in tumor area (Figure S5A), cortical and trabecular bone areas, or fibrotic and necrotic areas, except for an increase in the proportion of necrotic tumor area in mice receiving the combination treatment of abiraterone and prednisone (Figure S5B). 

### 3.5. Zoledronic Acid Inhibits Increased Bone Resorption Associated with the Combination of Radium-223, Abiraterone Acetate, and Prednisone

**Figure 6:**
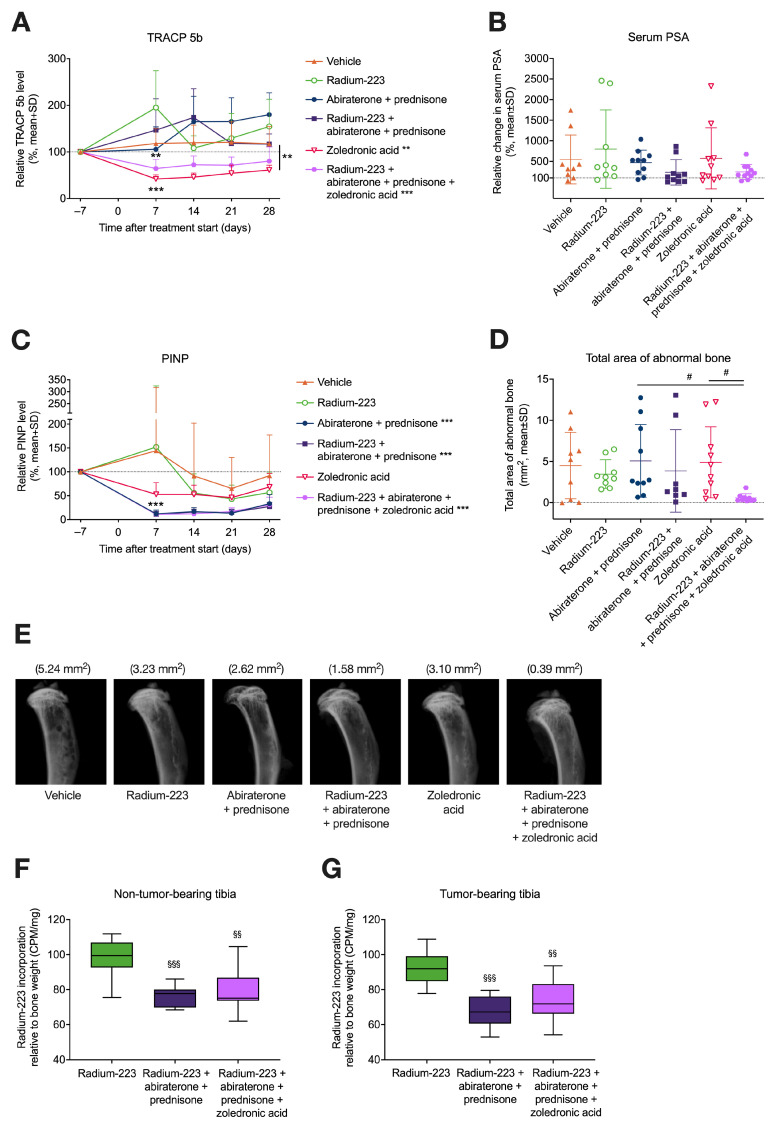
Zoledronic acid inhibits increased bone resorption associated with the combination of radium-223, abiraterone acetate, and prednisone. Blood samples were collected from the saphenous vein of LNCaP tumor-bearing mice (*n* = 9–12) treated with vehicle, radium-223 (330 kBq/kg, Q4Wx2), abiraterone (200 mg/kg, QD), prednisone (2.5 mg, 60-day release pellet), zoledronic acid (0.01 mg/kg, Q4Wx2) or their combinations. (**A**) TRACP 5b levels, (**B**) serum PSA levels at the end of the study, as well as (**C**) PINP levels, were measured by ELISA and normalized to pre-treatment baseline values. (**D**) Total area of abnormal bone in tumor-bearing tibiae as evaluated by ex vivo radiography. (**E**) Representative X-ray images of tumor-bearing tibiae at sacrifice. Total area of abnormal bone for each representative sample is indicated in parentheses. Radium-223 incorporation into (**F**) non-tumor-bearing and (**G**) tumor-bearing tibiae collected from mice treated with radium-223 monotherapy (*n* = 9), the combination of radium-223, abiraterone, and prednisone (*n* = 8) or the combination of radium-223, abiraterone, prednisone, and zoledronic acid (*n* = 11). The results are expressed as counts per minute (CPM) normalized to the weight of the bone sample. Statistical analyses for TRACP 5b (**A**) and PINP (**C**) were performed using mixed models and model contrasts: **, *p* < 0.01; ***, *p* < 0.001. Ex vivo radiography (**D**) and radium-223 incorporation (**F**,**G**) were analyzed using ANOVA and ANOVA contrasts: ^#^, *p* < 0.05, compared with radium-223, abiraterone, prednisone, and zoledronic acid combination; ^§§^, *p* < 0.01; ^§§§^, *p* < 0.001, compared with radium-223 monotherapy. The box plots (**F**,**G**) describe median, 25/75% quartiles, and the minimum and maximum values.

Next, we investigated whether zoledronic acid prevents the observed bone resorption (Figure 3) associated with the combination treatment of radium-223, abiraterone acetate, and prednisone in the intratibial LNCaP model. In Study 2, the combination of radium-223, abiraterone, and prednisone transiently increased TRACP 5b levels as well, but zoledronic acid as both monotherapy (*p* < 0.001) and as an addition to the combination treatment (*p* = 0.002) were able to decrease TRACP 5b levels after one week of treatment, indicating prevention of bone resorption in these groups (Figure 6A). Moreover, zoledronic acid monotherapy (*p* = 0.005) decreased TRACP 5b levels throughout the study when compared with vehicle (Figure 6A). However, as expected, zoledronic acid did not show any antitumor effects as monotherapy or as part of the combination treatment, as evidenced by the unaffected serum PSA levels in mice (Figure 6B). In line with Study 1, PINP levels were dramatically decreased in all prednisone-treated groups (*p* < 0.001), but zoledronic acid monotherapy had only a modest decreasing effect on PINP (Figure 6C). Compared with abiraterone and prednisone combination treatment and zoledronic acid monotherapy, the combination treatment of radium-223, abiraterone, prednisone, and zoledronic acid reduced the total area of abnormal bone in the tumor-bearing tibiae as measured by ex vivo radiography (Figure 6D–E). In fact, zoledronic acid, together with the combination of radium-223, abiraterone, and prednisone, inhibited abnormal bone growth in tumor-bearing bone more efficiently compared with other groups receiving radium-223. This confirms that supportive zoledronic acid treatment is not affecting the ability of radium-223 to inhibit abnormal bone growth. As previously shown in Figure 2C, the radium-223, abiraterone, and prednisone combination treatment reduced radium-223 incorporation into bone compared with the radium-223 monotherapy group. Concurrent administration with zoledronic acid did not weaken radium-223 incorporation either into non-tumor-bearing or tumor-bearing bone as compared with the combination of radium-223, abiraterone, and prednisone (Figure 6F–G).

## 4. Discussion

In this study, we investigated the mechanisms leading to increased risk of fractures associated with the combination treatment with radium-223, abiraterone, and prednisone observed in the ERA 223 clinical trial. To this end, we used the intratibial LNCaP mouse model mimicking prostate cancer with osteoblastic and mixed osteoblastic/osteolytic bone metastases. The changes in bone fragility observed in the ERA 223 trial could be induced by multiple factors, such as a combination of chronic androgenic deprivation by abiraterone together with prednisone administration, insufficient use of BPAs, and use of preventive fracture measures (weight-bearing exercises) as suggested by Spratt et al. [28].

Bone health management in patients with prostate cancer remains a major challenge due to bone loss induced by suppressed testosterone production by traditional androgen deprivation therapy (ADT) and various tumor-induced changes in bone in the areas of bone metastases. Testosterone deficiency induces severe bone-related AEs, particularly clinically relevant bone loss and fractures, due to reduced bone density and microarchitectural deterioration of bone [29,30]. Novel hormonal therapies for CRPC, such as abiraterone or enzalutamide, can impair bone health as well. In the first abiraterone phase 3 study (COU-AA-301), non-pathological fractures were almost twice as common in patients treated with the combination of abiraterone and prednisone (5.9% of patients) as compared with prednisone alone (2.3% of patients) [31]. A meta-analysis consisting of four phase 3 trials revealed that non-pathological fractures were observed in 10.2% of patients treated with enzalutamide compared with 4.4% of patients receiving placebo treatment [32]. Therefore, it seems evident that both abiraterone and enzalutamide can contribute to an increased risk of fractures, even in the absence of radium-223. However, we recently demonstrated that radium-223, in combination with enzalutamide, enhances antitumor efficacy without compromising the integrity of healthy bone in the intratibial LNCaP model [33].

In line with clinical outcomes observed in the ERA 223 study [7], the combination treatment with radium-223, abiraterone, and prednisone had no significant effect on tumor growth in this study, as determined by serum PSA. Transient increases in PSA levels were observed in the prednisone monotherapy group, but at the end of the experiment, the PSA levels did not differ between the treatment groups. The transient increase in serum PSA by prednisone may be due to a mutation of the androgen receptor (AR) in the LNCaP cells, which broadens the ligand binding specificity, leading to promiscuous binding of the AR [34]. In the prednisone combination treatment groups, radium-223 and abiraterone may prevent PSA production by killing cancer cells and inhibiting AR signaling, respectively. This could at least partially explain why the transient increase in PSA concentration on day 14 is only observed in the prednisone monotherapy group. The combination treatment showed no additive antitumor efficacy compared with radium-223 monotherapy, which may be due to the observed treatment-induced reduction of radium-223 incorporation into bone by abiraterone and/or prednisone. In contrast, we have previously shown that concomitant enzalutamide treatment with radium-223 did not affect radium-223 incorporation into bone in the intratibial LNCaP model [33]. In line with this observation, enhanced/synergistic antitumor effects were observed for radium-223 in combination with enzalutamide [33]. With regard to the radium-223, abiraterone, and prednisone combination treatment in clinic, the reduced radium-223 incorporation into bone metastases could be one explanation for why no improvements in overall survival or SRE-free survival were observed in the ERA 223 study [7], unlike in previous studies with radium-223 as monotherapy [3,35]. However, the ERA 223 study had certain limitations as patients were selected based on specific eligibility criteria that do not translate into routine clinical practice. Furthermore, bone mineral density was not assessed at baseline or during the study [7]. Moreover, the complex interplay of multiple factors present in the clinical setting, including variability in patient characteristics, duration of treatment, and treatment background, certainly contribute to the observed effects in the ERA 223 study. 

The transient effects seen in the levels of bone turnover biomarkers TRACP 5b, total ALP, and PINP indicate notable changes in bone remodeling immediately after the initiation of the radium-223, abiraterone, and prednisone combination treatment. In this study, the radium-223, abiraterone, and prednisone combination treatment specifically induced a transient increase of TRACP 5b levels in serum, showing an additive effect when compared with the combination of abiraterone and prednisone. As TRACP 5b is a sensitive marker for monitoring osteoclast-specific bone resorption [36], the results suggest that radium-223 can boost the bone resorptive effects of abiraterone and prednisone, leading to impaired bone remodeling and, thus, increase the risk of fractures. Total ALP levels were also transiently increased by abiraterone after one week of treatment. This is in line with previous studies, which have shown that total ALP levels typically are elevated in CRPC patients during the first 2–4 weeks of abiraterone treatment, commonly referred to “ALP flare” or “ALP bouncing”, only to subsequently decline to pre-treatment levels or further below [37]. It has been suggested that combination treatment with radium-223, abiraterone, and prednisone could promote radium-223 incorporation into non-tumor-bearing bone, leading to alpha radiation-mediated cell and tissue damage and, thus, non-pathological fractures [38]. The logical explanation for this suggested increased radium-223 incorporation would be higher osteoblastic activity in bone, induced by abiraterone-induced “ALP flare”. In contradiction, we observed reduced radium-223 incorporation in bones of mice treated with the radium-223, abiraterone, and prednisone combination treatment. However, in this case, radium-223 incorporation was measured two weeks after the peak of “ALP flare”.

In contrast to the increases observed in TRACP 5b and total ALP levels, a transient decrease in PINP levels was induced by prednisone-related treatments, suggesting that prednisone promotes catabolic bone metabolism, possibly through the inhibition of osteoblast activity. Based on the clinical findings of the ERA 223 study, the concomitant administration of radium-223 and prednisone has been hypothesized to induce synergistic inhibition of osteoblast activity, differentiation, and maturation, leading to increased bone fragility [39]. Prednisone could play a crucial role in this as glucocorticoids are known to reduce PINP levels [40,41], suppress osteoblast function, induce osteoblast apoptosis in vivo [42], and cause long-term changes in trabecular bone formation [43]. However, as there are several factors that might affect bone formation in response to the combination treatment with radium-223, abiraterone, and prednisone, it is possible that reduced PINP levels indicate the inhibition of osteoblast activity but do not linearly reflect the inhibition of new bone formation in the intratibial LNCaP model. Moreover, one must consider that the measured bone markers reflect both cancer-induced and treatment-induced changes in bone turnover, which complicates the interpretation of these bone turnover biomarker results. In addition, during the treatment, the mice are aging, and the normal growth of bones in young mice is slowing down, which also affects the bone marker levels and further complicates the interpretation of treatment effects on bone markers.

In agreement with the observed decrease in PINP levels, dynamic histomorphometry analyses revealed that the combination treatment of radium-223, abiraterone, and prednisone reduced bone formation in non-tumor-bearing bone. The indicators of bone formation, i.e., trabecular and periosteal mineralizing surface, were reduced in mice treated with either radium-223 or prednisone, respectively, when compared with the vehicle treatment. Only the radium-223, abiraterone, and prednisone combination treatment resulted in a reduction of both trabecular and periosteal mineralizing surfaces. It has been previously shown that radium-223 treatment reduces the number of osteoblasts in LNCaP xenografts [15], and a similar effect was observed in this study in non-tumor-bearing bone. However, the osteoclast number in non-tumor-bearing bone remained unaffected.

Despite the changes in bone turnover biomarkers or the reduction of bone formation assessed by dynamic histomorphometry, no structural or biomechanical changes were observed in the bone as determined by microCT and 3-point bending test analyses, respectively. In the ERA 223 study, out of all fracture types, osteoporotic fractures were the most common type of fractures induced by the combination treatment with radium-223, abiraterone, and prednisone [7]. Our microCT results showed that prednisone reduced the trabecular bone volume and trabecular thickness in non-tumor-bearing tibia but not in tumor-bearing tibia. This could partly explain the increased amount of non-pathological fractures in the ERA 223 study [7]. However, we observed no changes in bone volume or thickness associated with the radium-223, abiraterone, and prednisone combination treatment compared with the vehicle treatment. Overall, our data indicate that in the intratibial LNCaP model, the combination treatment with radium-223, abiraterone, and prednisone had no weakening effects on bone architecture or biomechanical properties that could explain the increased fragility observed in the clinic. In general, future studies using other models mimicking prostate cancer metastasized to bone, such as LuCaP 58 patient-derived xenograft model, which we have used previously [15], could facilitate the interpretation of the results obtained in the intratibial LNCaP model. Furthermore, it remains to be evaluated whether the reduced radium-223 incorporation to bone observed in the radium-223, abiraterone, and prednisone combination treatment group is due to prednisone, abiraterone, or their combination. 

Zoledronic acid given concomitantly with radium-223, abiraterone, and prednisone did not interfere with radium-223 antitumor efficacy or radium-223 incorporation into bone in the intratibial LNCaP model. Increased bone resorption induced by the radium-223, abiraterone, and prednisone treatment was inhibited by zoledronic acid, as indicated by lower TRACP 5b levels compared with the vehicle treatment. Zoledronic acid treatment in combination with radium-223, abiraterone, and prednisone resulted in smaller areas of tumor-induced abnormal bone formation when compared with zoledronic acid monotherapy or the combination of abiraterone and prednisone, but not with other treatments, including radium-223, suggesting that zoledronic acid itself does not inhibit tumor-induced abnormal bone growth/formation. In our previous study with a bone metastatic breast cancer xenograft model, radium-223 improved survival in mice with established bone metastases, and this survival benefit was evident with or without concurrent zoledronic acid treatment [44]. Accordingly, a recent clinical study demonstrated that zoledronic acid does not improve the overall survival of patients with metastatic CRPC treated with radium-223 [45].

Interestingly, in a recent retrospective study where radium-223 was given as monotherapy in second- or third-line treatment after the combination treatment of abiraterone and prednisone, a low risk of fractures was observed [46]. This clinical finding supports the possibility of underlying synergistic effects in the concomitant administration of radium-223, abiraterone, and prednisone, which can lead to an increase in non-pathological fractures. 

Based on our data and previous studies [15,33,44], we suggest the following mode of action for the radium-223, abiraterone, and prednisone combination treatment. Radium-223 treatment reduces osteoblast number and inhibits trabecular bone formation in non-tumor-bearing bones. Furthermore, prednisone inhibits osteoblast activity, as indicated by decreasing PINP levels, and inhibits periosteal bone formation in non-tumor-bearing bone. Moreover, bone resorption stimulated by both abiraterone and prednisone is enhanced by radium-223, which is demonstrated by a transient increase in TRACP 5b levels in the intratibial LNCaP model. As a result, bone remodeling is shifted towards bone resorption, as highlighted in the Graphical Abstract. Thus, our preclinical results obtained in the intratibial LNCaP model support the current practice that radium-223 is not indicated with abiraterone and prednisone, even for patients with mCRPC receiving BPAs, such as bisphosphonates and denosumab. Presumably, BPAs are able to prevent stimulated osteoclastogenesis and osteoclast activity but not the anti-osteoblastic effects of abiraterone and prednisone. Nevertheless, several species and model-related factors may limit the translational relevance of these findings and, thus, could also explain the lack of structural or biomechanical changes in the combination treatment groups. Studies on other preclinical models, for instance, with the LuCaP 58 patient-derived xenograft model, could provide valuable evidence on this matter. 

## 5. Conclusions

In conclusion, concurrent zoledronic acid treatment inhibits the bone resorption induced by the combination treatment of radium-223, abiraterone, and prednisone, possibly abolishing one of the mechanisms which may contribute to the increased fracture risk observed in the ERA 223 trial. The anti-osteoblastic effects of radium-223, in combination with the bone resorptive effects of abiraterone and prednisone, may lead to impaired bone remodeling with suppressed bone formation and, thus, increase the risk of non-pathological fractures. 

## Data Availability

The data presented in this study are available in this article and Supplementary Materials.

## Supplementary Materials

The following supporting information can be downloaded at: https://www.mdpi.com/article/10.3390/cancers15164115/s1 Figure S1: Relative body weight change in LNCaP tumor-bearing mice; Figure S2: Bone marker levels in the intratibial LNCaP model; Figure S3: Total, trabecular, and cortical bone volumes of tumor-bearing and non-tumor-bearing tibiae in the intratibial LNCaP model; Figure S4: Biomechanical quality of femoral shafts in the intratibial LNCaP model; Figure S5: Total tumor area and relative necrotic tumor area of tumor-bearing tibiae in the intratibial LNCaP model; Table S1: Dynamic and static histomorphometry parameters measured from non-tumor-bearing tibiae of LNCaP tumor-bearing mice.
