# Supplementary material for: Zoledronic Acid Prevents Bone Resorption Caused by the Combination of Radium-223, Abiraterone Acetate, and Prednisone in an Intratibial Prostate Cancer Mouse Model
Source: Cancers (Basel). 2023 Aug 15;15(16):4115. doi: 10.3390/cancers15164115 (PMC10452892; doi:10.3390/cancers15164115)
Supplement: Supplementary file 1 [file cancers-15-04115-s001.zip › cancers-2519296-supplementary.pdf]

SUPPLEMENTARY MATERIALS

Bone structure by microCT

The bone volume, cross-sectional dimensions, and microarchitecture of tumor-bearing and non-tumor-bearing tibiae of mice were measured by micro-computed tomography (microCT) and quantified using a SkyScan 1276 high-resolution microCT scanner (Bruker, Billerica, MA, USA) (1-3). *In vivo* microCT measurements for total bone volume were performed under isoflurane anesthesia when serum PSA reached over 0.14 ng/mL. *Ex vivo*, two separate volumes of interests (VOI) were analyzed in metaphyseal trabecular bone and one VOI in diaphyseal cortical bone. Reconstructed microCT images were prepared for one representative mouse in each study group.

Biomechanical quality of femoral shafts by 3-point bending test

The whole-bone biomechanical properties, including stiffness (N/mm), yield displacement (mm), maximum load (N), load at fracture (N), post-yield displacement (mm), and work-to-fracture (Nmm), were determined in femoral shafts by a 3-point bending test (4-8) using an Instron 3343 biomechanical testing system (Instron, Norwood, MA, USA). The longitudinal length of the femur was measured using a Vernier caliper prior to the biomechanical testing.

Bone labeling

For measuring dynamic histomorphometry parameters in bone, mice were injected with fluorescence dyes twice during the treatment period of the study. Bone labeling was performed with either oxytetracycline (20 mg/kg, i.p.) or alizarin (30 mg/kg, s.c.) seven days before sacrifice and with calcein green (10 mg/kg, s.c.) two days before sacrifice. The mice in the pre-treatment group were not injected. Oxytetracycline (Terramycin Vet solution, Zoetis Finland) and solid calcein green (#C0875; Sigma-Aldrich) were dissolved in 0.9% NaCl. Solid alizarin (#A3882; Sigma-Aldrich) was dissolved in 2% sodium bicarbonate. Subsequently, the final dosing solutions

containing 4 mg/mL of oxytetracycline, 2 mg/mL of calcein green, and 15 mg/mL of alizarin were sterile filtered.

Bone microarchitecture, bone formation, and cellular characteristics by histomorphometry

Static and dynamic histomorphometry for bone microarchitecture, bone formation, and cellular characteristics of non-tumor-bearing tibiae were analyzed using an OsteoMeasure7 histomorphometry system (OsteoMetrics, Decatur, GA, USA) (9,10). Non-tumor-bearing tibiae were dehydrated in EtOH, defatted in xylene and embedded in methyl methacrylate-based plastic. After the embedding, longitudinal sections were obtained from a standardized site of the proximal tibia using a fully motorized rotary microtome and a tungsten-carbide knife. Static trabecular bone parameters were determined from two 4-μm-thick sections stained in Masson-Goldner's Trichrome and dynamic trabecular bone parameters from two unstained 8-μm-thick sections. Cross-sectional cylinders were prepared from a standardized site of the tibial shaft in a transverse plane using a linear precision saw and a diamond blade. Both static and dynamic cortical bone parameters were determined from an unstained 200-μm-thick cylinder. All parameters were analyzed following the guidelines of the American Society for Bone and Mineral Research (ASBMR) (11). Reported parameters are listed in **Table S1**. As the oxytetracycline/calcein green bone double labeling was not successful in the majority of cortical bone samples but only the single label was observed, bone formation parameters requiring only the single label were analyzed from the cortical bone.

Tumor, fibrotic, and necrotic areas by histology

Tumor-bearing tibiae were cut from the diaphysis in order to save bone samples for cortical histomorphometry. The proximal end of the tibia was processed into plastic blocks. Longitudinal 4-μm tibia sections were obtained and stained with Masson-Goldner Trichrome as described above for the protocol of the histomorphometric

analyses. The slides were scanned using Pannoramic 250 Flash and Pannoramic 1000 slide scanners (3DHISTECH Ltd). Tumor area, cortical and trabecular bone areas, and fibrotic and necrotic areas were analyzed from both a standardized area (encompassing 5 mm from the articular surface) and the whole section using CaseViewer software version 2.3 (3DHISTECH Ltd). The tumor, fibrotic, and necrotic areas were indicated in the images by lining the areas manually, and the bone area was defined by color threshold.

REFERENCES

1. Bouxsein ML, Boyd SK, Christiansen BA, Guldberg RE, Jepsen KJ, Muller R. Guidelines for assessment of bone microstructure in rodents using micro-computed tomography. J Bone Miner Res **2010**;25(7):1468-86
2. van 't Hof RJ. Analysis of bone architecture in rodents using microcomputed tomography. Methods Mol Biol **2012**;816:461-76
3. Engelke K, Prevrhal S, Genant HK. Macro- and microimaging of bone architecture. In: John P. Bilezikian LGR, T. John Martin, editor. Principles of bone biology. San Diego, CA, USA: Academic Press; **2008**. p 1905-42.
4. Goodyear SR, Aspden RM. Mechanical properties of bone ex vivo. Methods Mol Biol **2012**;816:555-71
5. Jepsen KJ, Silva MJ, Vashishth D, Guo XE, van der Meulen MC. Establishing biomechanical mechanisms in mouse models: practical guidelines for systematically evaluating phenotypic changes in the diaphyses of long bones. J Bone Miner Res **2015**;30(6):951-66
6. Morgan EF, Bouxsein ML. Biomechanics of Bone and Age-Related Fractures. In: John P. Bilezikian LGR, T. John Martin, editor. Principles of Bone Biology. Third Edition ed: Academic Press; **2008**.
7. Peng Z, Tuukkanen J, Zhang H, Jamsa T, Vaananen HK. The mechanical strength of bone in different rat models of experimental osteoporosis. Bone **1994**;15(5):523-32
8. Turner CH, Burr DB. Basic biomechanical measurements of bone: a tutorial. Bone **1993**;14(4):595-608
9. Dempster DW. Histomorphometric analysis of bone remodeling. In: John P. Bilezikian LGR, T. John Martin, editor. Principles of bone biology. San Diego, CA, USA: Academic Press, San Diego; **2008**. p 447-63.
10. Erben RG, Glosmann M. Histomorphometry in rodents. Methods Mol Biol **2012**;816:279-303
11. Dempster DW, Compston JE, Drezner MK, Glorieux FH, Kanis JA, Malluche H*, et al.* Standardized nomenclature, symbols, and units for bone histomorphometry: a 2012 update of the report of the ASBMR Histomorphometry Nomenclature Committee. J Bone Miner Res **2013**;28(1):2-17

SUPPLEMENTARY FIGURES AND TABLES

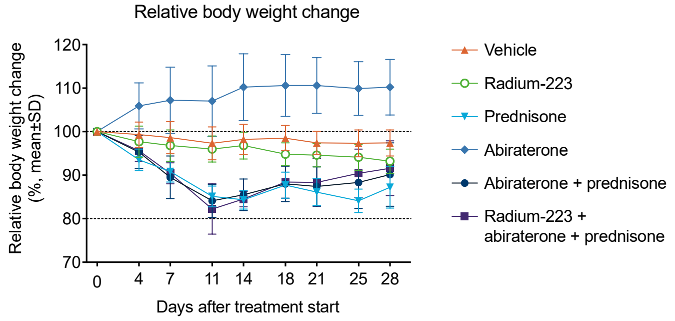

Figure S1. Relative body weight change in LNCaP tumor-bearing mice. Relative body weight (normalized to body weight at the start of treatment) in LNCaP tumor-bearing mice treated with vehicle, radium-223 (330 kBq/kg, Q4W x 2), prednisone (2.5 mg, 60-day release pellet), abiraterone (200 mg/kg, QD), or their combinations for 28 days

A

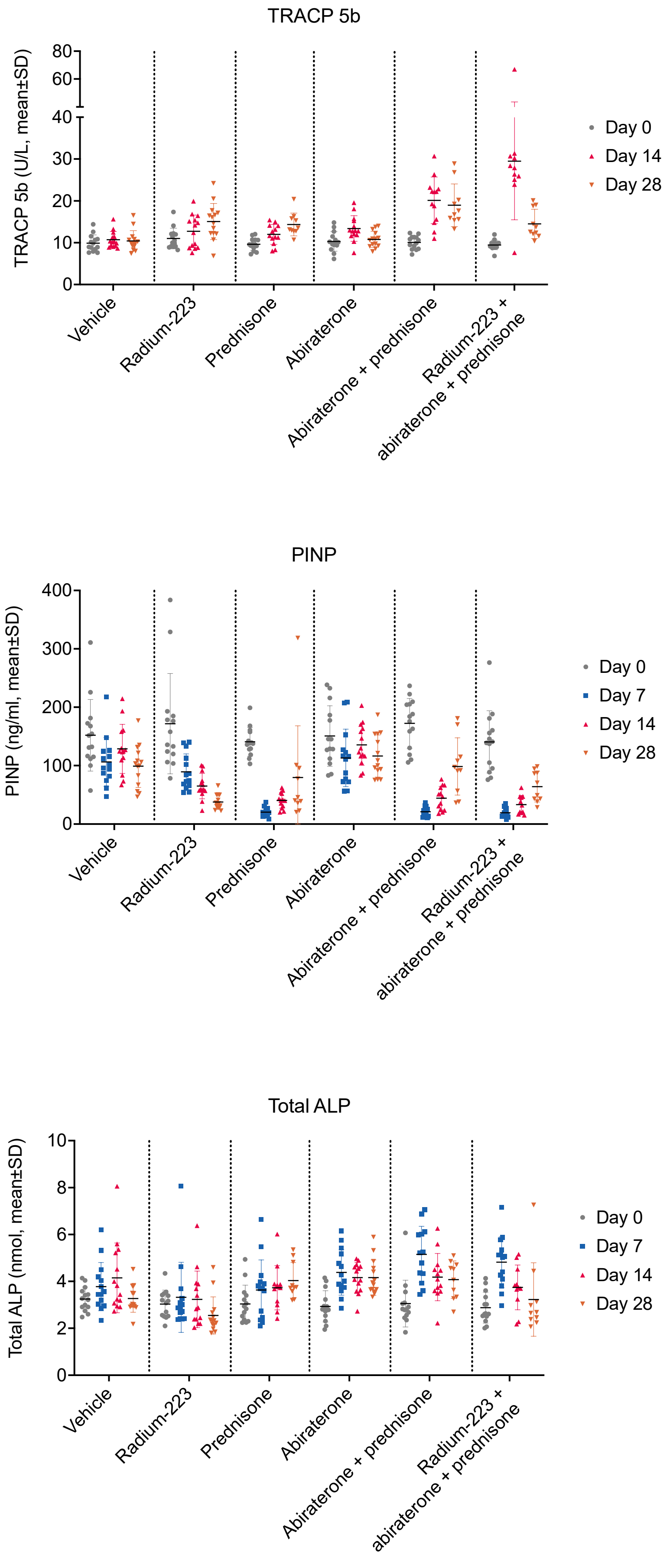
TRACP 5b
TRACP 5b (U/L, mean±SD)
80
60
40
30
20
10
0
Vehicle
Radium-223
Prednisone
Abiraterone
Abiraterone + prednisone
Radium-223 +
abiraterone + prednisone
Day 0
Day 14
Day 28

B

PINP
PINP (ng/ml, mean±SD)
400
300
200
100
0
Vehicle
Radium-223
Prednisone
Abiraterone
Abiraterone + prednisone
Radium-223 +
abiraterone + prednisone
Day 0
Day 7
Day 14
Day 28

C

Total ALP
Total ALP (nmol, mean±SD)
10
8
6
4
2
0
Vehicle
Radium-223
Prednisone
Abiraterone
Abiraterone + prednisone
Radium-223 +
abiraterone + prednisone
Day 0
Day 7
Day 14
Day 28

.

Figure S2. Bone marker levels in the intratibial LNCaP model. (**A**) TRACP 5b, (**B**) PINP, and (**C**) total ALP levels were measured in blood samples collected from the saphenous vein of LNCaP tumor-bearing mice (n=10–14) 7, 14 or 28 days after the initiation of treatment. The mice were treated with vehicle, radium-223 (330 kBq/kg, Q4Wx2), prednisone (2.5 mg, 60-day release pellet), abiraterone (200 mg/kg, QD), or their combinations

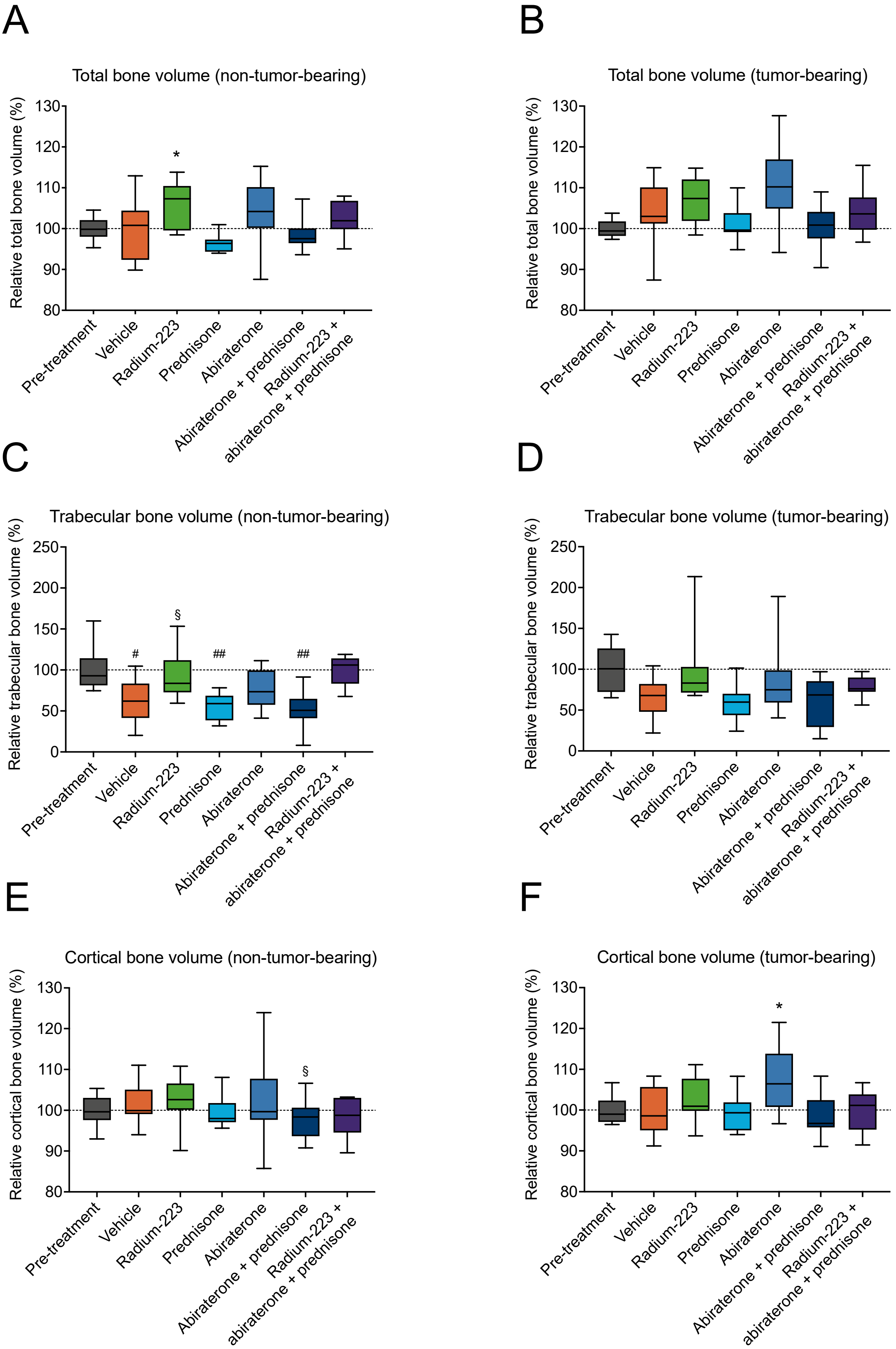

A
Total bone volume (non-tumor-bearing)
Relative total bone volume (%)
130
120
110
100
90
80
*
Pre-treatment
Vehicle
Radium-223
Prednisone
Abiraterone
Abiraterone + prednisone
Radium-223 + abiraterone + prednisone
B
Total bone volume (tumor-bearing)
Relative total bone volume (%)
C
Trabecular bone volume (non-tumor-bearing)
Relative trabecular bone volume (%)
250
200
150
100
50
0
#
§
##
##
D
Trabecular bone volume (tumor-bearing)
Relative trabecular bone volume (%)
E
Cortical bone volume (non-tumor-bearing)
Relative cortical bone volume (%)
§
F
Cortical bone volume (tumor-bearing)
Relative cortical bone volume (%)
*

Figure S3. Total, trabecular, and cortical bone volumes of tumor-bearing and non-tumor-bearing tibiae in the intratibial LNCaP model. Total (**A-B**), trabecular (**C-D**) and cortical bone (**E-F**) volume of non-tumor-bearing and tumor-bearing tibiae were quantified using a microCT scanner. Change in bone volume from randomization to sacrifice as normalized to values at randomization. The mice were treated with vehicle, radium-223 (330 kBq/kg, Q4W x 2), prednisone (2.5 mg, 60-day release pellet), abiraterone (200 mg/kg, QD), or their combinations. The box plots describe median, 25/75% quartiles, and the minimum and maximum values. Statistical analyses were performed using ANOVA or Kruskal-Wallis test and Dunn's test: *, $p<0.05$; compared with vehicle; $^{\#}$, $p<0.05$; $^{\#\#}$, $p<0.01$, compared with the combination of radium-223, abiraterone, and prednisone. "§" indicates groups in which one datapoint has been removed from the analysis as an outlier.

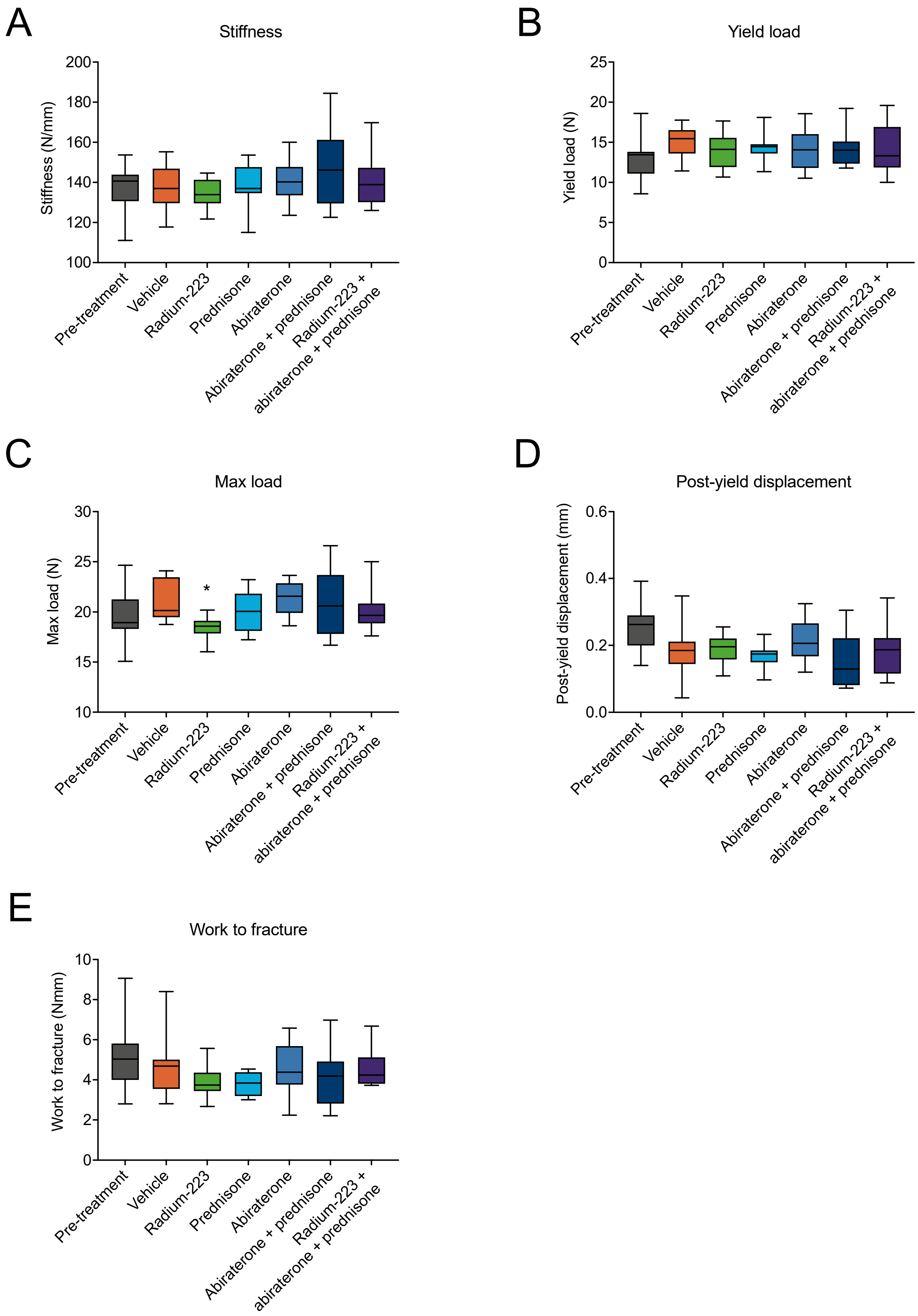
A
Stiffness
Stiffness (N/mm)
200
180
160
140
120
100
B
Yield load
Yield load (N)
25
20
15
10
5
0
C
Max load
Max load (N)
30
25
20
15
10
*
D
Post-yield displacement
Post-yield displacement (mm)
0.6
0.4
0.2
0.0
E
Work to fracture
Work to fracture (Nmm)
10
8
6
4
2
0
Pre-treatment
Vehicle
Radium-223
Prednisone
Abiraterone
Abiraterone + prednisone
Radium-223 + abiraterone + prednisone

Figure S4. Biomechanical quality of femoral shafts in the intratibial LNCaP model. The biomechanical quality of femoral shafts was measured by a 3-point bending test instrument, including the following biomechanical parameters: (**A**) stiffness, (**B**) yield load, (**C**) max load, (**D**) post-yield displacement and (**E**) work to fracture. The mice were treated with vehicle, radium-223 (330 kBq/kg, Q4W x 2), prednisone (2.5 mg, 60-day release pellet), abiraterone (200 mg/kg, QD), or their combinations. The box plots describe median, 25/75% quartiles, and the minimum and maximum values. Statistical analyses were performed using ANOVA: *, $p<0.05$, compared with vehicle.

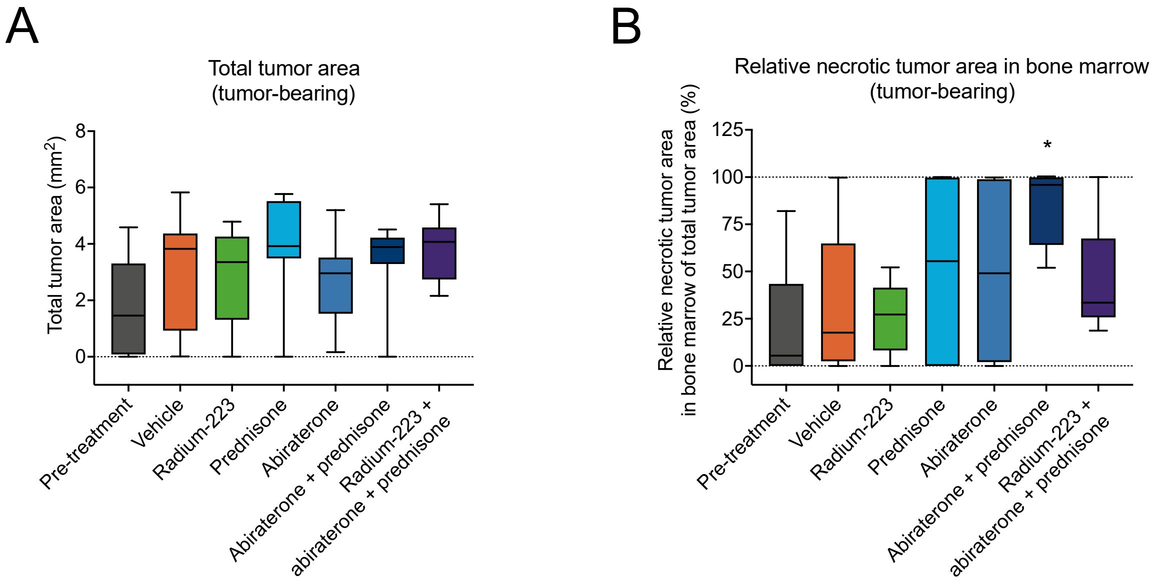

Figure S5. Total tumor area and relative necrotic tumor area of tumor-bearing tibiae in the intratibial LNCaP model. Histological analysis of (**A**) total tumor area and (**B**) relative necrotic tumor area in bone marrow (BM) normalized to total tumor area. The mice were treated with vehicle, radium-223 (330 kBq/kg, Q4W x 2), prednisone (2.5 mg, 60-day release pellet), abiraterone (200 mg/kg, QD), or their combinations for 28 days. The box plot describes median, 25/75% quartiles, and the minimum and maximum values. Statistical analyses were performed using Kruskal-Wallis and Dunn's test: *, $p<0.05$, compared with vehicle.

Table S1: Dynamic and static histomorphometry parameters measured from non-tumor-bearing tibiae of LNCaP tumor-bearing mice

Method	Parameter	Standard unit
Dynamic	Trabecular mineralizing surface	MS/BS (%)
	Periosteal mineralizing surface	MS/BS (%)
Static	Osteoblast number	N.Ob/Bp (mm-1)
	Osteoclast number	N.Oc/Bp (mm-1)

Bp, bone perimeter; BS, bone surface; MS, mineralizing surface; N.Ob, osteoblast number; N.Oc, osteoclast number
